# Novel Decorations at Wakefield Mental Hospital

**Published:** 1914-01-03

**Authors:** 


### Novel Decorations at Wakefield Mental Hospital.

It is frequently stated that the Christmas spirit is dying out. However far this may be true in a general sense, it certainly does not apply to Wakefield Mental Hospital. For many weeks attendants, nurses, and patients have been busily engaged in preparing decorations to adorn the wards at the festive season. The tissue-paper, which is supplied to each ward, is transformed with infinite pains and skill into chains, festoons, and flowers. By these means the wards, many of which are structurally gloomy, are changed into a veritable fairyland. The decorations are in no sense stereotyped, each ward exhibiting an individuality of its own. The general standard is so high that it is difficult to select the most striking features for comment. In the Acute Female Hospital, Ward 1, under the inspiration of Charge Nurse Hasker, with its masses of red roses, purple and heliotrope clematis, and blossom, presents an almost perfect colour scheme. Ward 3, thanks largely to Charge Nurse Lambert, is noteworthy for the exquisite character of the flowers, especially the roses and Madonna lilies. In the chronic block, Ward 32, Charge Nurse Young, is especially deserving of notice, and it has been transformed into a bower of floral beauty.

The male side strikes a bold and original note, and its various ingenious details repay a careful scrutiny. In Ward 18, Charge Attendant Bennett has painted some beautiful water-colours on the glass panels. Ward 2, Charge Attendant Pilling, is perhaps the most striking in the whole building, and it reminds one of a fairy grotto. Ward 35, Charge Attendant Bramham, is especially remarkable for a beautifully decorated ceiling. In the Acute Male Hospital, Ward 4, Charge Attendant Dixon, is most tastefully decorated, credit being especially due to the great skill of one of the patients. Ward 3, under the supervision of Charge Attendant Wright, has also produced extremely original decorative work.

Great credit is due to the staff for the time, trouble, and, in many instances, money they have expended in giving pleasure to over two thousand patients, who are prevented by illness from being among their friends at this season of the year.

